# Safety and complications of medical thoracoscopy in the management of pleural diseases

**DOI:** 10.1186/s12890-019-0888-5

**Published:** 2019-07-10

**Authors:** Yun-Yan Wan, Cong-Cong Zhai, Xin-Shan Lin, Zhou-Hong Yao, Qing-Hua Liu, Ling Zhu, De-Zhi Li, Xi-Li Li, Ning Wang, Dian-Jie Lin

**Affiliations:** Department of Respiratory Medicine, Shandong Provincial Hospital affiliated to Shandong University, Shandong University, No. 324 Jingwuweiqi Road, Jinan, Shandong Province 250021 People’s Republic of China

**Keywords:** Medical thoracoscopy, Pleural diseases, Safety, Complication

## Abstract

**Background:**

Medical thoracoscopy is considered an overall safe procedure, whereas numbers of studies focus on complications of diagnostic thoracoscopy and talc poudrage pleurodesis. We conduct this study to evaluate the safety of medical thoracoscopy in the management of pleural diseases and to compare complications in different therapeutic thoracoscopic procedures.

**Methods:**

A retrospective study was performed in 1926 patients, 662 of whom underwent medical thoracoscopy for diagnosis and 1264 of whom for therapeutic interventions of pleural diseases. Data on complications were obtained from the patients, notes on computer system, laboratory and radiographic findings. Chi-square test was performed to compare categorical variables and Fisher’s exact test was used for small samples.

**Results:**

The mean age was 51 ± 8.4 (range 21–86) years and 1117 (58%) were males. Diagnostic procedure was taken in 662 (34.4%) patients, whereas therapeutic procedure was taken in 1264 (65.6%) patients. Malignant histology was reported in 860 (44.6%) and 986 (51.2%) revealed benign pleural diseases. Eighty patients (4.2%) were not definitely diagnosed and they were considered as unidentified pleural effusion. One patient died during the creation of artificial pneumothorax, and the causes of death were supposed as air embolism or an inhibition of phrenic motoneurons and circulatory system. Complication of lung laceration was found in six patients (0.3%) and reexpansion pulmonary edema was observed in two patients (0.1%). Higher incidence of prolonged air leak was observed in bulla electrocoagulation group, in comparison with pleurodesis group. Moreover, pain and fever were the most frequently complications in pleurodesis group and cutaneous infection in entry site was the most frequently reported complication in pleural decortication of empyema group.

**Conclusions:**

Medical thoracoscopy is generally a safe and effective method, not only in the diagnosis of undiagnosed pleural effusions, but also in the management of pleural diseases. Mastering medical thoracoscopy well, improving patient management after the procedure and attempts to reduce the occurrence of post-procedural complications are the targets that physicians are supposed to achieve in the future.

## Background

Thoracoscopy was first used in 1865 by Francis Richard Cruise, a physician from Ireland [1] and in 1866 Dr. Samuel Gordon reported its use to examine the pleural space in a girl with empyema [2]. In 1998, for the first time, semirigid thoracoscope was evaluated as a new instrument for the examination of thoracic cavity [3]. Compared with surgical thoracoscopy, medical thoracoscopy (MT) requires local anaesthesia under conscious sedation and usually one or two points of entry with a result of less invasive and less expensive [4].

Medical thoracoscopy is initially used for diagnosing exudative pleural effusion [5], and it has been proved to be superior to fluid cytology and ‘blind’ closed needle pleural biopsy [6–8] with an accuracy of 98–100% [9]. There are several therapeutic applications of MT, including pleurodesis, treatment of infected pleural space, forceps lung biopsy and sympathectomy [10, 11].

When performed by experienced physicians under local anesthesia and moderate sedation, medical thoracoscopy is considered an overall safe procedure [12–14]. Complications of MT can be divided into major and minor complications. According to a comprehensive study, major complications (pneumonia, hemorrhage, empyema, bronchopleural fistula, port site tumor growth, postoperative pneumothorax or air leak) occurred in 86/4736 cases (1.8%). Minor complications (fever, minor haemorrhage, subcutaneous emphysema, operative skin site infection, atrial fibrillation, hypotension during procedure) occurred in 177/2411 procedures (7.3%) [14]. However, few clinical trials ever reported complications of MT in the management of pleural diseases.

The aim of present research is to evaluate the safety of MT and to compare incidence of complications in different therapeutic thoracoscopic procedures.

## Methods

### Patients

A retrospective study was performed in 1926 patients who underwent MT for diagnostic and therapeutic purposes in the department of Respiratory Medicine in Shandong Provincial Hospital affiliated to Shandong University from 1992 to 2017.

Pre-thoracoscopic investigations were conducted as the following items: pleural fluid analysis, chest X-ray and CT, chest ultrasonography (US), ECG, and routine laboratory investigations. Exclusion criteria: severe COPD and respiratory insufficiency, inability to tolerate partial or complete unilateral collapse of the lung, severe contralateral lung or pleural involvement, uncontrollable cough, a fused pleural space with extensive adhesions..

Patients’ data were obtained from the hospital’s Patient Administration System Database. The following clinical variables were recorded: data on demographics, physical, laboratory and radiologic findings, instruments (semi-rigid or rigid medical thoracoscopy), thoracoscopic findings and therapeutic procedures, histopathologic results, microbiological results, complications of MT, and treatment evaluation of patients.

### Medical Thoracoscopy

Medical thoracoscopy was conducted with a rigid thoracoscope (OLYMPUS; GERMANY) or a semirigid thoracoscope (OLYMPUS MEDICAL SYSTEMS CORP., Tokyo, Japan). Patients were placed in the lateral decubitus position with conscious sedation using diazepam or benzodiazepine. The lateral area of chest was sterilized and draped, 10 mL of 2% lidocaine (Silver Lake Shiyao Pharmaceutical Co.Ltd.; Yuncheng, Shanxi Province, China) with 0.5 mg of epinephrine [GrandPharma (China) Co., Ltd.; Wuhan, Hubei Province] was administered to the selected intercostal space for local anesthesia. Oxygen via nasal cannula was administered with 3-4 L/min to maintain SpO2 > 90%. During the entire procedure, blood pressure, cardiac rhythm, and oxygen saturation were monitored by continuous electrocardiograph and percutaneous oximetry monitoring. Thoracoscopy was performed with single or double entry ports [15, 16]. The single port, in the mid-axillary line between the 4th and 7th intercostal spaces, was commonly used for diagnostic procedure and pleural biopsy, while double ports were used for pleural decortication of empyema, to facilitate adhesiolysis, to control bleeding, to perform pleurodesis or bulla electrocoagulation. The second port was chosen depending on intrathoracic condition, usually in line with the first entry and located one or two intercostal spaces superior or inferior to the first.

Biopsies were taken from parietal pleura for histopathology and microbiological examinations. When MT was used for pleurodesis, 3–5 g of talc was insufflated into the pleural cavity with a catheter and bulb syringe. Bulla electrocoagulation was commonly implemented in patients with recurrent pneumothorax [17]. When extensive adhesions were found under thoracoscopy and precluded lung re-expansion, adhesiolysis with biopsy forceps or electrothermal forceps was performed. In addition, thoracoscopy was done early in the course of empyema where fluid could be easily evacuated and lysis of fibrinopurulent adhesions can be completely operated, while in some cases with a thick pleural peel or a complicated multiloculated pleural space, pleural decortication under thoracoscope can be performed as well [11].

A 28F chest tube was inserted at the end of the procedure through the same or the first entry port and chest drain was connected to an underwater drainage bottle. The chest tube can be removed if complete lung re-expansion was confirmed on CXR or fluid drainage was less than 100 mL/24 h.

### Statistical analysis

The descriptive analysis was expressed as frequency and mean ± SD. Chi-square test was performed to compare categorical variables and Fisher’s exact test was used for small samples. A *p*-value < 0.05 was considered statistically significant. Bonferroni correction was made for adjusting the effects of multiple comparisons of the variables. Data were analyzed using SPSS 19.0 statistical software (SPSS Inc., Chicago, IL, USA).

## Results

One thousand nine hundred and twenty-six patients were involved in the study with a mean age of 51 ± 8.4 years (range 21–86). Characteristics of the patients were shown in Table 1. In patients who had bilateral pleural effusion, MT was performed on the side with larger pleural effusion. The diagnosis of empyema was made by microbiological and biochemical examinations of pleural fluid before MT.

**Table 1:** Patients characteristics for medical thoracoscopy (*n* = 1926)

Variable	Value
Age (years)	
Mean (±standard deviation)	51 (±8.4)
Range	21–86
Gender	
Male	1117 (58.0)
Female	809 (42.0)
Affected side	
Left	770 (40.0)
Right	1102 (57.2)
Bilateral	54 (2.8)
Operation of medical thoracoscopy	
Diagnostic procedure	662 (34.4)
Therapeutic procedure	1264 (65.6)
Pleurodesis	358 (28.3)
Adhesiolysis	517 (40.9)
Pleural decortication of empyema*	125 (9.9)
Bulla electrocoagulation	264 (20.9)
Types of instruments	
Rigid medical thoracoscopy	1586 (82.4)
Semi-rigid medical thoracoscopy	340 (17.6)

*Cases of empyema were diagnosed before medical thoracoscopy;

Values are presented as No. (%) unless otherwise indicated

Diagnostic outcomes were represented in Table 2. Eighty patients were not definitely diagnosed before they discharged from hospital and they were considered as unidentified pleural effusion.

**Table 2:** Diagnostic outcomes of medical thoracoscopy (*n* = 1926)

Diagnosis outcomes	Number (%)
Benign	986 (51.2)
Pneumothorax	264 (26.8)
Parapneumonic effusion	366 (37.1)
Tuberculous pleurisy	317 (32.1)
Other benign pleural diseases	39 (4.0)
Malignant	860 (44.6)
Mesothelioma1	48 (17.2)
Pleural metastasis	712 (82.8)
Unidentified pleural effusion	80 (4.2)

Values are presented as No. (%) unless otherwise indicated

Table 3 revealed complications during and after MT. One 63-year-old male patient diagnosed as empyema died during the creation of pneumothorax before thoracoscopy. The patient was given artificial respiration with oxygen; chest compression was performed; coramine and adrenaline were injected intravenously. These efforts were of no avail and the patient died after 30 min. No procedure-related deaths happened in other cases. Hemolytic pleural fluid drainage exceeded 1000 ml/10 h in one patient who was converted to thoracic surgery. In addition, 401 patients (20.8%) had a temperature > 37.4 °C and temperature dropped to normal level with physical cooling or non-steroidal anti-inflammatory drugs (NSAIDS). Of the 137 cases of cutaneous infection in entry site, none resulted in severe empyema or sepsis. Moreover, re-expansion pulmonary edema was reported in two patients with large to moderate amount of pleural effusion. Both patients suffered from refractory cough and dyspnea, and CXR on bedside showed diffuse opacity of the right hemithorax. Dyspnea was improved by high-flow oxygen and IV diuretics, with CXR at 48 h after MT showing disappearance of the previously observed changes.

**Table 3:** Complications of medical thoracoscopy (*n* = 1926)

Complications	Number (%)
Major complications	
Mortality	1 (0.1)
Lung laceration	6 (0.3)
Bleeding	7 (0.4)
Reexpansion pulmonary edema	2 (0.1)
Prolonged air leak	9 (0.5)
Mediastinal emphysema	2 (0.1)
Minor complications	
Subcutaneous emphysema	62 (3.2)
Pain	749 (38.9)
Fever	401 (20.8)
Cutaneous infection in entry site	137 (7.1)

Values are presented as No. (%) unless otherwise indicated

**Fig. 1:**
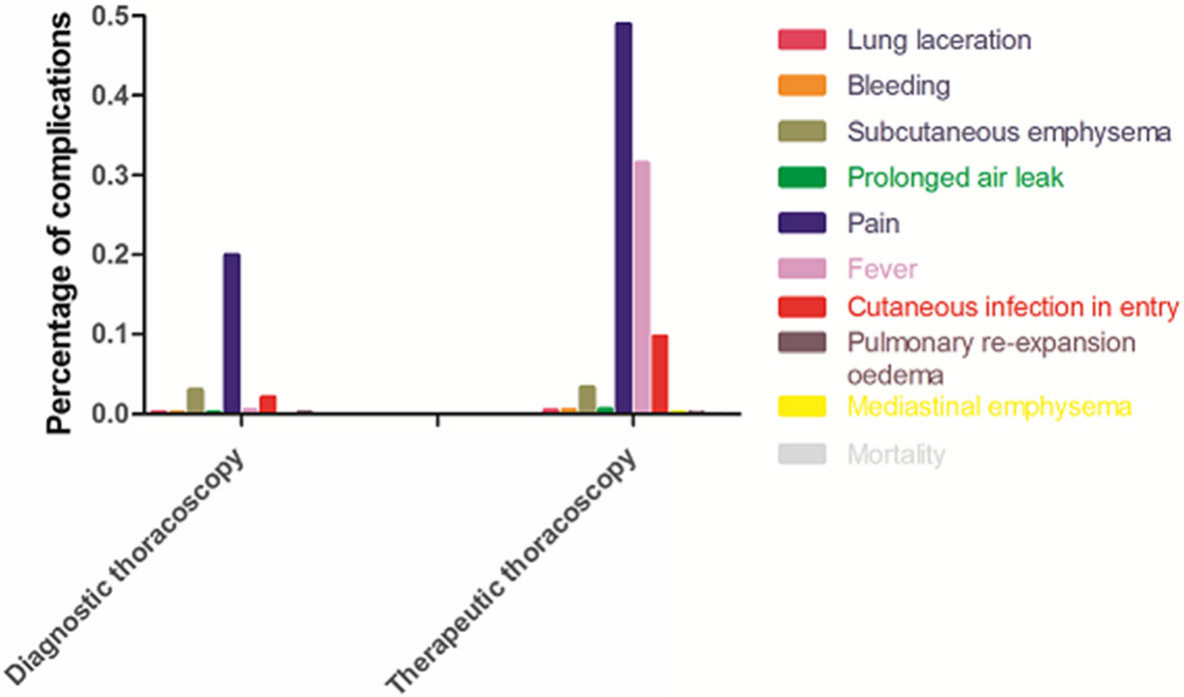
Distribution of complications in diagnostic and therapeutic thoracoscopy. In diagnostic thoracoscopy group, complications of pain, subcutaneous emphysema and cutaneous infection in entry site were more common; in therapeutic thoracoscopy group, complications of pain, fever and cutaneous infection in entry site were more common

Distribution of complications in diagnostic and therapeutic thoracoscopy was illustrated in Fig. 1. Incidence of pain, fever and cutaneous infection in entry site was higher in patients who underwent therapeutic thoracoscopy, according to the data mentioned in Table 4.

**Table 4:** Comparison of complications between diagnostic and therapeutic thoracoscopy (*n* = 1926)

Complications	Diagnostic thoracoscopy (*n* = 662)	Therapeutic thoracoscopy (*n* = 1264)	*P*-values
Major complications			
Mortality	0	1 (0.1)	1.0
Lung laceration	1 (0.2)	5 (0.4)	0.628
Bleeding	1 (0.2)	6 (0.5)	0.47
Pulmonary reexpansion oedema	1 (0.2)	1 (0.1)	1.0
Prolonged air leak	1 (0.2)	8 (0.6)	0.262
Mediastinal emphysema	0	2 (0.2)	0.549
Minor complications			
Subcutaneous emphysema	20 (3.0)	42 (3.3)	0.722
Pain	132 (19.9)	617 (48.8)	<0.001
Fever	3 (0.5)	398 (31.5)	<0.001
Cutaneous infection in entry site	14 (2.1)	123 (9.7)	<0.001

Values are presented as No. (%) unless otherwise indicated

**Fig. 2:**
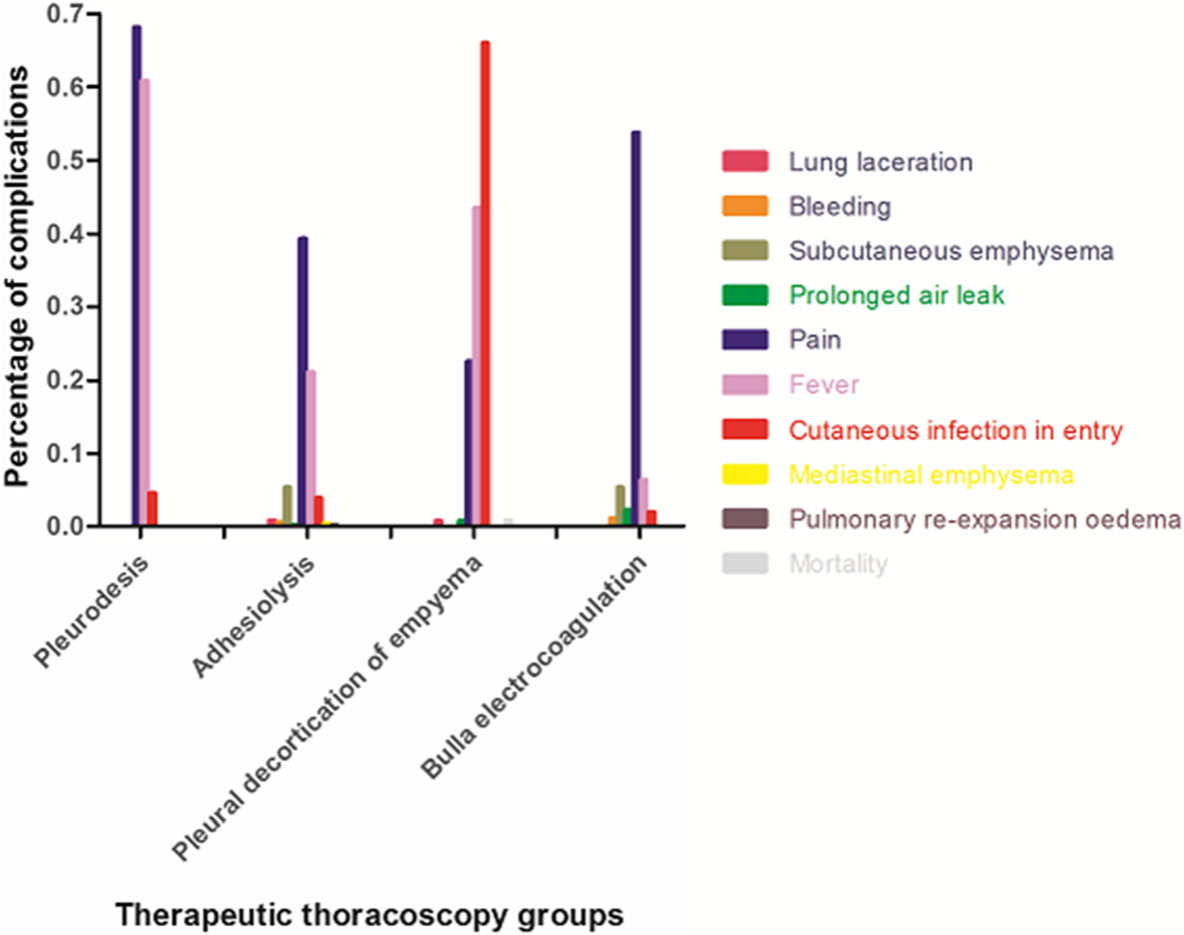
Distribution of complications in therapeutic thoracoscopy groups. In pleurodesis group, complications of pain, fever and cutaneous infection in entry site were more common; in adhesiolysis group, complications of pain, fever and subcutaneous emphysema were more common; in pleural decortication of empyema group, complications of cutaneous infection in entry site, fever and pain were more common; in bulla electrocoagulation group, complications of pain, fever and subcutaneous emphysema were more common

Figure 2 described complications of different therapeutic thoracoscopy groups. Comparing with other therapeutic methods, complication of subcutaneous emphysema was more common in adhesiolysis and bulla electrocoagulation groups (Table 5). Higher incidence of prolonged air leak was observed in bulla electrocoagulation group, in comparison with pleurodesis group. Furthermore, pain and fever were the most frequently complications in pleurodesis group and cutaneous infection in entry site was the most frequently reported complication in pleural decortication of empyema group.

**Table 5:** Comparison of complications in therapeutic thoracoscopy group (*n* = 1264)

Complications	Therapeutic thoracoscopy				*P*-values
	Pleurodesis (*n* = 358)	Adhesiolysis (*n* = 517)	Pleural decortication of empyema (*n* = 125)	Bulla electrocoagulation (*n* = 264)	
Major complications					
Mortality	0	0	1 (0.8)	0	0.099
Lung laceration	0	4 (0.8)	1 (0.8)	0	0.158
Bleeding	0	3 (0.6)	0	3 (1.1)	0.185
Pulmonary re-expansion oedema	0	1 (0.2)	0	0	1.0
Prolonged air leak	0	1 (0.2)	1 (0.8)	6 (2.3)	0.002 *
Mediastinal Emphysema02	0	2 (0.4)	0	0	0.597
Minor complications					
Subcutaneous emphysema	0	28 (5.4)	0	14 (5.3)	<0.001*
Pain	244 (68.2)	203 (39.3)	28 (22.4)	142 (53.8)	<0.001*
Fever	218 (60.9)	109 (21.1)	54 (43.2)	17 (6.4)	<0.001*
Cutaneous infection in entry site	16 (4.5)	20 (3.9)	82 (65.6)	5 (1.9)	<0.001*

Values are presented as No. (%) unless otherwise indicated

*Significance after adjustment with Bonferroni correction (*P* <0.0083)

## Discussion

Medical thoracoscopy refers to a invasive procedure to be commonly performed as a definitive diagnostic method [14] in undiagnostic exudative pleural effusion, as well as to be widely used in the management of pleural diseases. Many authors claim that MT is a safe procedure with low mortality, in patients who underwent MT for diagnostic thoracoscopy and talc poudrage pleurodesis [18–20]. In our comprehensive research, we found therapeutic interventions of MT increased risks of adverse actions.

One patient, who was diagnosed as empyema, died when artificial pneumothorax was being established. A general examination of the cardiovascular and central nervous systems revealed no abnormalities before MT. While 260 ml atmospheric air was slowly insufflated into pleural cavity, pleural pressure rapidly increased and respiratory-cardiac arrest occurred in the patient. Because of refusal of autopsy, the cause of death is not definitely confirmed. In light of the data collected and the representation of this patient’s death, the underlying mechanism is suspected. First of all, it is assumed that air embolism is the cause of death. Chest ultrasonography before MT revealed extensive adhesions in the pleural cavity and rapidly increased intrathoracic pressure could induce tearing of pleural adhesions, which resulted in rupture of veins on pleura. Large amount of air entered the ruptured veins and the bubbles could pass from intercostal veins through the azygos vein system to the superior vena cava, and could kill the patient by producing air locks in the right ventricle. In addition, there is one more point that a range of nerve reflexes stimulated by rapidly increased intrathoracic pressure result in respiratory depression and cardiac arrest. It has been reported in rabbits that increased pressure on the thoracic pleura induces a marked inhibition of phrenic motoneurons and a sympathetic outflow reduction of the circulatory system [21]. We suspect that increased intrathoracic pressure in the patient reduced both the amplitude of phrenic nerve discharge and systolic and diastolic pressure, and resulted in the inhibition of inspiratory activity and cardiac function. To prevent these risks, it is safer to be performed by trained experienced pulmonologists and measurements of lung function are implemented before the procedure. During the creation of pneumothorax, operator should feel and observe negative respiratory fluctuations on the manometer until pleural pressure nears zero, or 800–1000 ml air has been insufflated. Pressure of air insufflation never exceeds 10 cmH2O, and inflation should be stopped if the patient is suffering from discomfort or resistance to insufflation is experienced. By this means, air embolism, cardiac disturbances or tearing of pleural adhesions would be avoided. Nevertheless, establishment of artificial pneumothorax in patients with extensive pleural adhesions is a paramount challenge that needs cautious consideration.

Lung laceration is an important complication which happened in 6 patients in whom artificial pneumothorax was not established. All of them occurred during introduction of the trocar and did not exceed 1 cm in length. During the entire procedure, no air leakage was detected and slight blood streamed from the site of the laceration. All the cases were neglected and no complications happened after MT. If the length of lung laceration exceeds 1 cm or excessive bleeding and air leakage are discovered, the following treatments can be performed by calling cardiothoracic surgeon and application of absorbable mesh and fibrin glue over the laceration under MT. Establishment of artificial pneumothorax a few hours or 1 day before MT can prevent lung laceration which was confirmed in our study. However, if there is enough pleural fluid, it is safe to introduce a blunt trocar into the thoracic cavity without prior creation of pneumothorax [22]. Chest ultrasonography and CT can be very helpful to identify pleural adhesions in chest wall and locate an appropriate entry site for thoracoscopy [9]. In our study, both chest ultrasonography and CT scan were done in the 6 cases with lung lacerations and a blunt trocar was used, the main causes were probably to be vigorous maneuver during introducing the trocar and absence of artificial pneumothorax. Furthermore, in this study, four of the six patients (66.7%) were in adhesiolysis group. It indicates that extensive adhesions in pleural cavity increase the risk of lung laceration when introducing a trocar, and location with chest US or CT before MT is not a guarantee of security. What degree of adhesions is insufficient for MT? What is the selection for patients with extensive adhesions, VATS or other pleural procedures? These crucial issues should be resolved in the future.

Re-expansion pulmonary edema (RPE) is a rare and potentially lethal complication and it occurred in two patients in our study. With regard to the first patient, a volume of 2000 ml pleural effusion was aspirated from pleural cavity during MT, and collapsed right lung reexpanded rapidly. Although exact pathogenesis of RPE is still not fully understood, some pathogenic factors for RPE have been reported, such as rapid reexpansion, airway obstruction, longer duration of lung collapse, decreased surfactant activity and increased pulmonary vascular permeability due to injuries to the pulmonary micro-vessels [23]. Regarding to the second patient with moderate amount of pleural effusion, the causes of RPE were considered as longer duration of lung collapse and rapid reexpansion after adhesiolysis. Re-expansion pulmonary edema is usually seen in ipsilateral lung but contrlateral or bilateral cases also have been reported [23]. Treatments of RPE include high-flow oxygen and IV diuretics. Other intensive therapies, such as circulation support, steroid administration, and mechanical ventilation, are required in severe cases of RPE [24, 25]. Repeated aspirations of air or pleural fluid to facilitate lung re-expansion before MT and use of antibechic after the procedure can prevent occurrence of RPE.

With respect to developing bleeding and chest pain, massive hemorrhage usually occurred when mechanical lysis of adhesions was performed in patients with pneumothorax. Under MT, abundant and thick blood vessels were observed in adhesion zones between the visceral and parietal pleura. We are conducting experiments to study structure and origination of the blood vessels. In patients with pneumothorax, adhesiolysis with electrothermal forceps is more favourable, instead of mechanical lysis. Pain was the most common complication in this study and can be well tolerated by most of patients. Severe pain usually happened when pleurodesis was conducted during MT and sometimes transient attack of hypotension happened due to violent pain. The procedure should be stopped and blood pressure can be elevated to normal range without medicine treatment. Blood pressure, heart rate and oxygen saturation should be monitored during pleurodesis, especially when the procedure is performed in patients with hypovolemia or cardiac insufficiency.

Prolonged air leak was more common in bulla electrocoagulation group. It is probably related to destruction of emphysematous bullae, and it is advised that bulla electrocoagulation combined with pleurodesis would reduce incidence of prolonged air leak.

In addition, the current study found that pleurodesis group had the highest incidence of fever, which was probably in relation to a systemic inflammation inducing by intrapleural insufflation of sclerosing agents. Steroids and NSAIDS could reduce the quality of pleurodesis [26–29], therefore physical cooling is recommended. With respect to developing cutaneous infection in entry site, it can be modified by improving patient management after the procedure, including dressing change regularly and the use of intravenous antibiotic.

One strengthen of the current research is that a large size of study population is investigated, in which 1926 patients are included. On the other hand, we have to admit that there are some limitations in our research. This is a retrospective study of practice and outcome, and perspective design is under consideration. Follow-up does not continue after patients discharge from hospital, therefore late complications are not represented in the study.

## Conclusions

In conclusion, when medical thoracoscopy is performed by experienced physicians, MT under local anesthesia is generally a safe and effective method for the diagnostic and therapeutic procedures. Mastering medical thoracoscopy well, improving patient management after the procedure and attempts to reduce the occurrence of post-procedural complications should be encouraged.

## Data Availability

The datasets used during the current study are available from the corresponding author on reasonable request. Due to the privacy policy of patients, A truncated dataset after eliminating all potentially identifiable features may be provided on an individual request basis.

## Abbreviations

COPD: Chronic obstructive pulmonary disease
CT: Computed tomography
CXR: Chest X-ray
ECG: Electrocardiogram
IV: Intravenous
MT: Medical thoracoscopy
NSAIDS: Non-steroidal anti-inflammatory drugs
RPE: Re-expansion pulmonary edema
US: Ultrasonography
VATS: Video-assisted thoracoscopic surgery
